# Next-generation mapping: a novel approach for detection of pathogenic structural variants with a potential utility in clinical diagnosis

**DOI:** 10.1186/s13073-017-0479-0

**Published:** 2017-10-25

**Authors:** Hayk Barseghyan, Wilson Tang, Richard T. Wang, Miguel Almalvez, Eva Segura, Matthew S. Bramble, Allen Lipson, Emilie D. Douine, Hane Lee, Emmanuèle C. Délot, Stanley F. Nelson, Eric Vilain

**Affiliations:** 1 Department of Human Genetics, David Geffen School of Medicine, University of California, Los Angeles, CA 90095 USA; 2 Department of Pediatrics, David Geffen School of Medicine, University of California, Los Angeles, CA 90095 USA; 3 Department of Pathology and Laboratory Medicine, David Geffen School of Medicine, University of California, Los Angeles, CA 90095 USA; 4 Center for Genetic Medicine Research, Children’s National Health System, Children’s Research Institute, Washington, DC, 20010 USA

**Keywords:** Next-generation mapping, Duchenne muscular dystrophy, Bionano, Structural variants, DMD, Optical mapping, Nanochannel

## Abstract

**Background:**

Massively parallel DNA sequencing, such as exome sequencing, has become a routine clinical procedure to identify pathogenic variants responsible for a patient’s phenotype. Exome sequencing has the capability of reliably identifying inherited and de novo single-nucleotide variants, small insertions, and deletions. However, due to the use of 100–300-bp fragment reads, this platform is not well powered to sensitively identify moderate to large structural variants (SV), such as insertions, deletions, inversions, and translocations.

**Methods:**

To overcome these limitations, we used next-generation mapping (NGM) to image high molecular weight double-stranded DNA molecules (megabase size) with fluorescent tags in nanochannel arrays for de novo genome assembly. We investigated the capacity of this NGM platform to identify pathogenic SV in a series of patients diagnosed with Duchenne muscular dystrophy (DMD), due to large deletions, insertion, and inversion involving the *DMD* gene.

**Results:**

We identified deletion, duplication, and inversion breakpoints within *DMD*. The sizes of deletions were in the range of 45–250 Kbp, whereas the one identified insertion was approximately 13 Kbp in size. This method refined the location of the break points within introns for cases with deletions compared to current polymerase chain reaction (PCR)-based clinical techniques. Heterozygous SV were detected in the known carrier mothers of the DMD patients, demonstrating the ability of the method to ascertain carrier status for large SV. The method was also able to identify a 5.1-Mbp inversion involving the *DMD* gene, previously identified by RNA sequencing.

**Conclusions:**

We showed the ability of NGM technology to detect pathogenic structural variants otherwise missed by PCR-based techniques or chromosomal microarrays. NGM is poised to become a new tool in the clinical genetic diagnostic strategy and research due to its ability to sensitively identify large genomic variations.

**Electronic supplementary material:**

The online version of this article (doi:10.1186/s13073-017-0479-0) contains supplementary material, which is available to authorized users.

## Background

Although Sanger sequencing is still widely used to sequence fragments of > 1 Kbp in length, massively parallel sequencing has emerged and now dominates the global market for sequencing due to its comprehensiveness, supported by increasingly cheap price and fast turnaround times. This is, in part, due to the improvements made in imaging, microengineering, and informatics techniques that enable acquisition of larger amounts of clean data from next-generation sequencing (NGS). This has allowed for an ever-expanding compendium of pathogenic single-nucleotide mutations for rare Mendelian genetic diseases (Online Mendelian Inheritance in Man). In the last several years, exome sequencing has successfully entered the clinical realm; however, only about 30% of cases are solved currently, implying that much of the genetic variation remains undetected [1, 2].

Unlike exome sequencing, whole-genome sequencing (WGS) is capable of identifying single nucleotide variants (SNVs), insertions/deletions, and copy number variants not only in exons, but also in non-coding regions of the genome. This allows for identification of variants affecting gene regulation, which currently has limited clinical use due to inability to interpret most variants’ effect on the open reading frame of potential disease genes. In addition, structural variation detection using WGS data presents challenges in highly repetitive genomic regions.

The major platform for NGS utilizes flowcells covered with millions of surface-bound oligonucleotides that allow parallel sequencing of hundreds of millions of independent short reads (100–300 bp) randomly selected from the human genome. The resulting reads oversample the diploid genome and are typically aligned to a reference genome for variant discovery. As the average library fragment size is 300–400 bp in length, structural variants (SV) can be challenging to observe. This is evident from the large number of SV calling programs. There are more than 40 programs designed to call SV using different approaches such as read-depth, read-pair, split-read methods, or combined [3] with each method having limitation and thus resulting in not one tool being able to survey all SVs. Number of SVs detected, false discovery rate, and sensitivity rate are estimated to range widely with low concordance rate even for the most commonly used programs [4, 5]. While short-read WGS can identify SV when the quality of read mapping is high, there are regions of the genome where SV breakpoints reside within repetitive sequences that are difficult to uniquely map. In clinical practice, it remains that the most common method for detecting large insertions or deletions is currently chromosomal microarrays (CMA). However, CMAs cannot detect balanced translocations or inversions and miss many genomic events < 30 Kbp.

A method that is useful in conjunction with WGS is genome mapping, which utilizes high molecular weight DNA labeled at specific sequence sites that accurately represent much larger fragments of the genome. These very long Mb-size fragments allow for the construction of scaffolds for sequence assembly into the two haploid genomes of an individual, facilitating direct examination of larger structural variants that would be difficult to observe with short-read sequencing methods [6]. As it creates a more complete map of an individual’s diploid genome, next-generation mapping (NGM) allows detection of translocation and inversion breakpoints, large insertions and deletions in the genome, and more complex SV.

However, NGM is an emerging tool that still needs to prove its value within the clinical genetic diagnostic practice. Bionano Genomics has developed a platform for NGM that images very long double-stranded fragments of DNA (dsDNA) nicked at specific sites to enable fluorescent tagging. The fluorescent tags are read efficiently by molecular combing within nanochannels. NGM has already been used for de novo assemblies of newly sequenced genomes and demonstrated that it can facilitate accurate construction of the whole genomes of individual species and for diploid human individuals [7]. The potential of this technology to sensitively identify SV may offer substantial advantages over current clinical diagnostic practice. However, due to its novelty and unproven track record in clinic, we sought to validate the ability of NGM to observe large SV in a cohort of patients diagnosed with Duchenne muscular dystrophy (DMD). DMD is an X-linked recessive muscular dystrophy that affects about one in 5000 male newborns. It is characterized by progressive loss of skeletal muscle function, heart failure, and pulmonary failure. The disease is caused by mutations in *DMD*, which encodes the dystrophin protein at Xp21. The 2.5-Mbp *DMD* gene, the largest gene in humans, is transcribed to a 14-Kbp mRNA with 79 exons. The *DMD* gene is one of the most common targets of de novo and consequential mutation in the genome. A study of over 7000 mutations in *DMD* showed that 86% of all mutations were large deletions of  ≥ 1 exon [8]. Here, we selected DMD patients referred to the UCLA Center for Duchenne Muscular Dystrophy. All of the probands in our cohort were known to carry multiexonic deletion or insertion mutations in *DMD* or, in one case, a large inversion that disrupted the *DMD* open reading frame (Table 1). We sought to determine if NGM was capable of identifying these large structural variants present in DMD probands as well as identify the carrier status in the mothers.

**Table 1:** Cohort of patients diagnosed with Duchenne muscular dystrophy (DMD)

Sample ID	Identifier	Detection method	SV identified	SV size (bp) by NGM	Coverage by NGM
CDMD1003	Proband	PCR	Hemizygous deletion (exons 46–51)	–182,665	72x
CDMD1155	Proband	MLPA	Hemizygous deletion (exons 48–54)	–224,364	104x
CDMD1156	Proband	MLPA	Hemizygous deletion (exons 49–50)	–59,771	74x
CDMD1159	Proband	MLPA	Hemizygous deletion (exon 52)	–45,839	90x
CDMD1131	Proband	PCR, MLPA	Hemizygous deletion (exons 45–partial 51)	–250,092	118x
CDMD1132	Mother	aCGH	Heterozygous deletion (exons 45–51 [carrier])	–249,994	96x
CDMD1157	Proband	MLPA	Hemizygous deletion (exons 46–51)	–184,882	85x
CDMD1158	Mother	N/A	Unknown before NGM; Not a carrier of exons 46–51 deletion	N/A	80x
CDMD1163	Proband	aCGH	Hemizygous duplication (exons 3–4)	+12,968	87x
CDMD1164	Mother	N/A	Unknown before NGM; carrier of exons 3–4 duplication	+12,857	158x
CDMD1187	Proband	PCR, MLPA, RNA-seq, WGS	Hemizygous inversion (exons 38–end)	5.1 Mb	90x

Cases with SV in *DMD* are shown. The “detection method” column describes methods used to identify affected exons of *DMD*. The “+” and “-” signs in the “SV size (bp) by NGM” column represent gain or loss of DNA material, respectively. The last column describes the effective genome coverage (defined as the total amount of the data produced in base pairs divided by the genome size [3.2 Gbp in the case of humans] and multiplied by molecule-to-reference map rate (typical range 55–85%).

*PCR* polymerase chain reaction, *RNA-seq* RNA sequencing, *WGS* whole-genome sequencing, *MLPA* multiplex ligation-dependent probe amplification, *aCGH* microarray-based comparative genomic hybridization

## Methods

We used the nanochannel-based NGM technology developed by Bionano Genomics to assemble a physical map of the human genome for identification of large insertions, deletions, translocations, and inversions.

### High molecular weight DNA isolation

High molecular weight DNA was extracted both from fresh (<5 days old) and frozen (– 80 °C) whole blood. DNA extraction was performed following the manufacturer’s guidelines (PlugLysis, Bionano Genomics, USA). RBC lysis solution (Qiagen) was used to lyse red blood cells and pellet white blood cells. The white blood cells were re-suspended in cell suspension buffer (Bio-Rad) and embedded into agarose plugs (CHEF Genomic DNA Plug Kit, Bio-Rad) to lessen fragmentation of long DNA molecules during the overnight lysis at 50 °C using a 16:1 ratio of lysis buffer (Bionano Genomics, USA) and Puregene Proteinase K (Qiagen). The plugs were washed with Tris-EDTA buffer and digested at 43 °C with GELase (Epicentre). Extracted high molecular weight DNA was purified from digested materials/enzymes via drop dialysis using Millipore membrane filters (EMD Millipore, USA) placed on Tris-EDTA buffer. DNA quantifications were carried out using Qubit dsDNA assay kits with a Qubit 3.0 Fluorometer (ThermoFisher Scientific).

### DNA labeling/chip loading

**Fig. 1:**
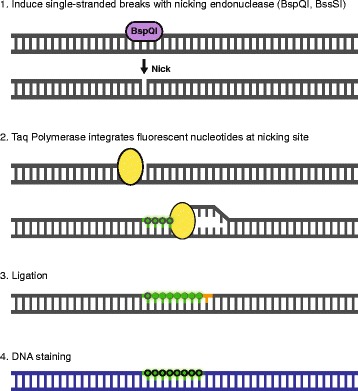
DNA labeling for NGM. The DNA labeling workflow is divided into four consecutive steps. First, the high molecular weight DNA is nicked with an endonuclease of choice that introduces single strand nicks throughout the genome. Second, Taq polymerase recognizes these sites and replaces several nucleotides with fluorescently tagged nucleotides added to the solution. Third, the two ends of the DNA are ligated together using DNA ligase. Fourth, the DNA backbone is stained with DNA Stain

**Fig. 2:**
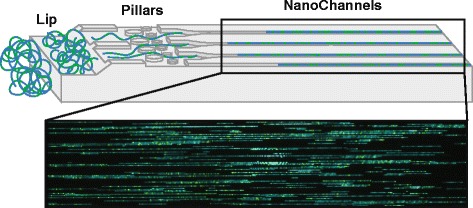
Irys/Saphyr chip nanochannel structure and DNA loading. The labeled dsDNA is loaded into two flowcells of either Irys or Saphyr chips. The applied voltage concentrates the coiled DNA at the lip (*left*). Later, DNA is pushed through pillars (*middle*) to uncoil/straighten, then into nanochannels (*right*). DNA is stopped and imaged in the nanochannels. *Blue* = staining of DNA backbone, *green* = fluorescently labeled nicked sites

DNA labeling consists of four sequential steps (Fig. 1) and was performed using the IrysPrep Reagent Kit (Bionano Genomics). Depending on the amount of coverage needed and the type of chip used, 300/600/900 ng of purified high molecular weight DNA was nicked with nicking endonucleases Nt.BspQI or Nb.BssSI (New England BioLabs/Bionano Genomics) in 10X Buffer 3 (Bionano Genomics) at 37 °C for 2 h. The nicked DNA was then labeled with 10X Labeling Mix containing fluorophore-labeled nucleotides using Taq polymerase (NEB) at 72 °C for 1 h before being repaired with Taq ligase (NEB) and IrysPrep Repair Mix, NAD+, and 10X Thermopol buffer at 37 °C for 30 min. DNA backbone was stained for visualization and size identification with IrysPrep DNA stain, 5X DTT, and 4X flow buffer overnight at 4 °C (Bionano Genomics). Labeled DNA was loaded on Irys chip and run for 24 h (Fig. 2). In the chip, the sample is run through a low-voltage electric field. DNA is first concentrated in a gradient region (lip) before being pushed through a pillar region, needed for DNA linearization before entering the nanochannel arrays. The fluorescently labeled DNA molecules are imaged sequentially across nanochannels by the Irys/Saphyr instrument producing thousands of high-resolution images of individual DNA molecules that are then used for genome assembly and variant calling. To achieve the necessary effective coverage of 70x (determined sufficient by Bionano internal validations) for accurate identification of structural variants, 4–6 Irys chips were run per endonuclease, each at 30 cycles. One Saphyr chip was sufficient to generate needed coverage for two enzymes (achieved in 30 cycles).

### De novo assembly

Genome assembly was performed using IrysView/IrysSolve software solutions provided by Bionano Genomics. The raw TIFF images of labeled long DNA molecules were converted to BNX files containing DNA backbone, nicked sites, and quality score information for each molecule/label. The conversion was accomplished via AutoDetect software (Bionano Genomics). Due to the large size of the raw data that is acquired in the form of TIFF images, we opted to store only BNX files. Assembly of the genome using BNX files and further structural variation detection was performed using pipelines generated by Bionano Genomics [9]. De novo assembly was performed using Bionano’s custom assembler software program based on the Overlap-Layout-Consensus paradigm (binary tools version 6119 and assembly pipeline version 6005). Pairwise comparison of all DNA molecules was done to create a layout overlap graph, which was then used to create the initial consensus genome maps. By realigning molecules to the genome maps (Refine-B *P* value 10^–11^) and by using only the best match molecules, label positions were refined and chimeric joins were removed. Next, during an extension step, the software aligned molecules to genome maps (Extension *P* value 10^–11^), and extended the maps based on the molecules aligning past the map ends. Overlapping genome maps were then merged using a Merge *P* value cutoff of 10^–15^. These extension-and-merge steps were repeated five times before a final refinement was applied to all genome maps (Refine Final *P* value 10^–11^).

During the extension step, the software identified clusters of molecules that aligned to genome maps with end alignment gaps of size > 30 Kbp (i.e. > 30 Kbp of one side of the molecules did not align), selected out these molecules and re-assembled them. In addition, the final refinement step searched for clusters of molecules aligned to genome maps with internal alignment gap of size < 50 Kbp, in which case the genome maps were converted into two haplotype maps. The extend-and-split function is essential to identify large allelic differences and to assemble across loci with segmental duplications, whereas the refinement haplotype function can find smaller differences.

### Structural variant calling

SV were called based on the alignment profiles between the de novo assembled genome maps against the public Genome Reference Consortium GRCh37 human assembly. If the assembled map did not align contiguously to the reference, but instead was broken into two alignments, then a putative structural variation was identified. We required an alignment cutoff of *P* value < 10^–12^ to identify the best-aligned locations. Significant discrepancies in the distance between adjacent labels or the number of unaligned labels between adjacent aligned labels (outlier *P* value 3 × 10^–3^) indicated the presence of an insertion (defined as a gain of genetic material in a form of duplications, triplications, amplifications, etc.) or a deletion (defined as a loss of genetic material). For small gain-of-material events, there may not be enough nick sites to identify the genomic origin of the abnormal material. Hence, almost all smaller events with gain of genetic material are called insertions. Genome maps whose alignments were in opposite orientations on the same chromosome indicated the presence of inversion breakpoints. Maps aligning to different chromosomes or aligning over 5 Mbp apart on the same chromosome suggested inter-chromosomal and intra-chromosomal translocations, respectively.

### Validation of SV via quantitative polymerase chain reaction (qPCR)

Validation of a newly identified insertion was performed using qPCR. The primer sequences used are detailed in (Additional file 1: Table S1). Primers were designed using primer design software Primer3 [10]. DNA was quantified using QuBit HS (Invitrogen) for dsDNA and a total of 2 ng of DNA was used per sample for qPCR reaction. qPCR was carried out in quadruplicates and duplicates using Syber Green-based SensiFAST™ SYBR No-ROX Kit (Bioline, UK) by DNA Engine Opticon® 2 real-time PCR detection system from Bio-Rad Laboratories (BioRad, USA). Reaction conditions were as follows: 95 °C for 10 min, then 40 cycles of 95 °C for 15 s, 60 °C for 10 s, and 72 °C for 15 s.

## Results

**Fig. 3:**
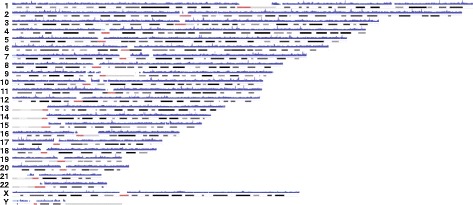
Visualization of the human genome coverage using NGM. Chromosome 1-22,X,Y are represented by G-banding patterns. The *red shading* represents centromere locations. Horizontal *blue shading* represents regions where long native-state DNA molecules have been aligned using the Bionano NGM platform

**Fig. 4:**
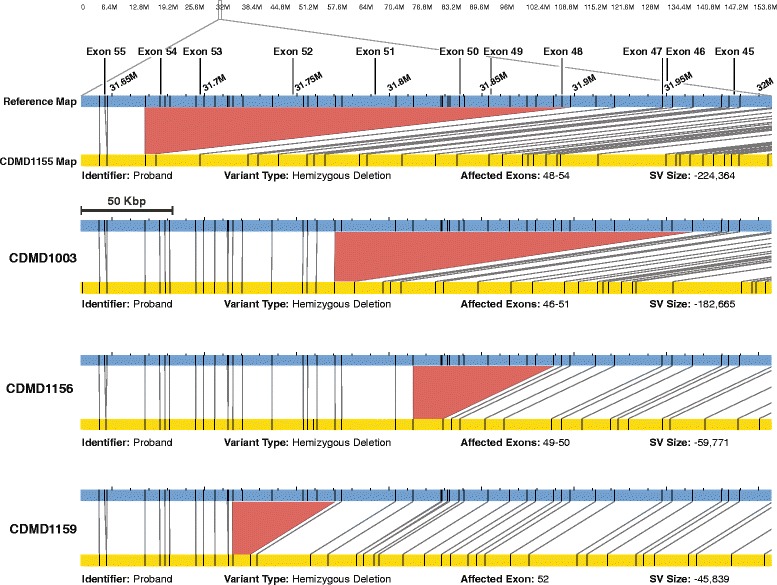
Deletions identified in four DMD probands. For each case, the *blue bar* represents the reference X chromosome. The *yellow bar* represents the sample map generated based on long molecule assembly of the patient’s genome. The *black vertical lines* indicate Nt.BspQI endonuclease cut sites and corresponding matches between reference (*blue*) and sample (*yellow*) genomes. The lines between reference and assembled map show alignment of the two maps. The *red area* indicates the deletion where reference (*blue*) endonuclease sites are missing from the assembled map (*yellow*). The locations of the *DMD* exons are indicated at the top of the figure with *vertical lines*. Below each map, information such as size and type of the SV and deleted exons can be found

We performed NGM on a cohort of eight affected DMD individuals (six with deletions, one with an insertion, and one with an inversion) and three biological mothers, one of whom was a known carrier of a pathogenic deletion in *DMD* (Table 1). Long DNA molecule representation throughout the genome was present at all known regions except at centromeres, acrocentric chromosomes, and long arm of the Y chromosome due to lack of presence of unique sequences (Fig. 3). Genetic diagnosis of DMD is most often achieved by PCR and multiplex ligation-dependent probe amplification (MLPA) of all 79 exons of *DMD*. Exonic sequence mutation analysis utilizes Sanger sequencing of PCR amplicons generated from each of the 79 exons, whereas MLPA uses probe hybridization and amplification to assay for deletions or duplications in the gene. In our DMD cohort, five singleton cases received a clinical diagnosis based on either PCR or MLPA. This type of commonly used deletion/duplication analysis of *DMD* does not provide an accurate positioning of the intronic breakpoints or the size of DNA that is deleted or inserted, only indicating the exons that are affected. Unlike MLPA, NGM technology is potentially capable of more accurately identifying the location of intronic breakpoints in the gene, which may become important as gene editing strategies emerge for DMD [11]. Using NGM we identified all previously known structural variants in the DMD cohort (Table 1). In addition, the method is capable of identifying both single (CDMD1159) and multiple exon deletions (e.g. CDMD1003; Fig. 4). The resolution of the breakpoints is limited to endonuclease nicking site density in a given region; higher density provides more accurate estimates. With a single enzyme the resolution of DNA breakpoints is in the range of 5–10 Kbp in size; however, it is possible to gain higher accuracy with the use of a second endonuclease, decreasing the uncertainty of breakpoint location from 5–10 Kbp to 1.5–3 Kbp [9].

**Fig. 5:**
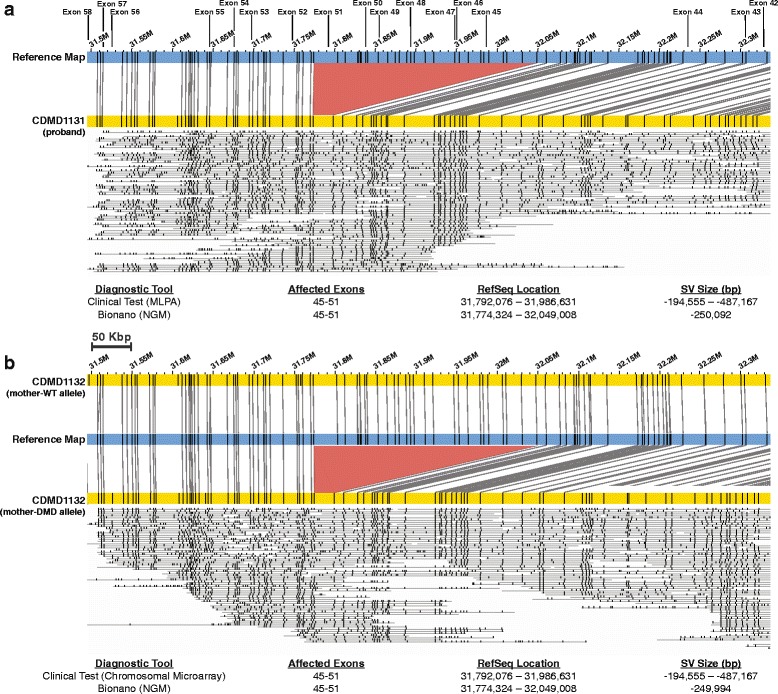
NGM identified a hemizygous and heterozygous multi-exon deletion in a DMD patient and his biological mother, respectively. **a** Hemizygous deletion in the patient. *Top*: visual representation of the deletion (*red*) between the reference (*blue*) and patient (*yellow*) maps. *Middle*: representation of long molecules used to construct the sample maps. *Bottom*: Ref-seq locations on the X chromosome indicating possible size of the deletion based on MPLA and size identified using the NGM platform. **b** Heterozygous deletion in the biological mother. *Top*: The normal wild type allele (*yellow*) can be seen above reference (*blue*) where all nicking sites align to reference map. This is in contrary to the second allele (*yellow*) containing the deletion shown below the reference (*blue*) map. Maps were generated using Nt.BspQI nicking endonuclease

We then tested whether NGM was capable of identifying heterozygous deletion/insertion status in the carrier mothers of DMD patients. We performed NGM on three DMD duos (proband and mother) to determine if the SV identified in the child was observed in the mother. CDMD1131, a proband, had a large pathogenic deletion in *DMD* spanning exons 45–51 (exon 51 partially present) that had been identified clinically by MLPA. The mother (CDMD1132) of this patient is heterozygous for this deletion as determined by chromosomal microarray. NGM testing of this duo confirmed the previous diagnosis of the proband (Fig. 5a) and the carrier status of the mother (Fig. 5b). In Fig. 5 a and b we can see that there are no adjacent flanking nicking sites close to exon 51 making it difficult to identify that part of the exon 51 is present as reported by MLPA. This is a major limitation of the method where the actual breakpoint could be between the two adjacent nicking sites to either side of exon 51.

**Fig. 6:**
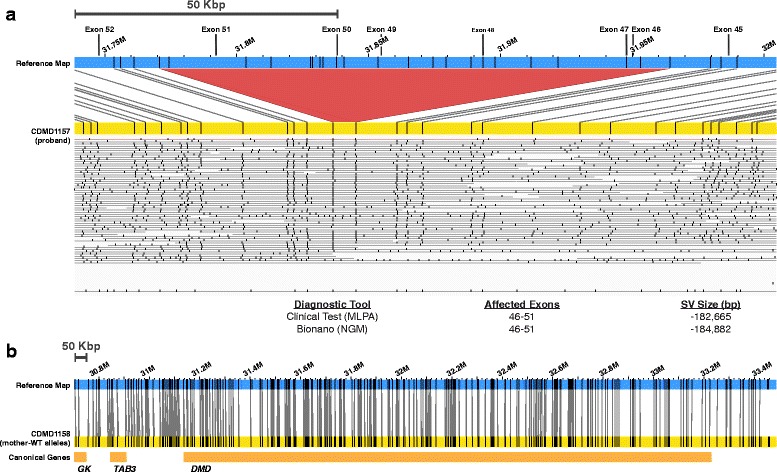
NGM identified a hemizygous multi-exon deletion in a DMD patient that was not present in the biological mother. **a**, **b** *Top*: visual representation of the sample allele in *yellow* (**a** patient; **b** mother) compared to the reference (*blue*). The de novo deletion is shown in *red*. **a** *Middle*: the *lines* below the patient’s contig represent the long molecules used to construct the sample map. *Bottom*: Ref-seq locations on the X chromosome indicating possible size of the deletion based on MPLA and size identified using the NGM platform. **b** *Bottom*: location of Ref-Seq genes in the X chromosome within the shown region. Maps were generated using Nt.BspQI nicking endonuclease

Proband CDMD1157 was also diagnosed clinically with a *DMD* deletion spanning exons 46–51; however, the carrier status of the mother (CDMD1158) was unknown. NGM identified a 185-Kbp deletion containing exons 46–51 in the proband (Fig. 6a) confirming the clinical diagnosis. NGM also showed that the mother was not a carrier of the same deletion or other SV in the *DMD* gene (Fig. 6b) indicating that this mutation occurred de novo in CDMD1157.

**Fig. 7:**
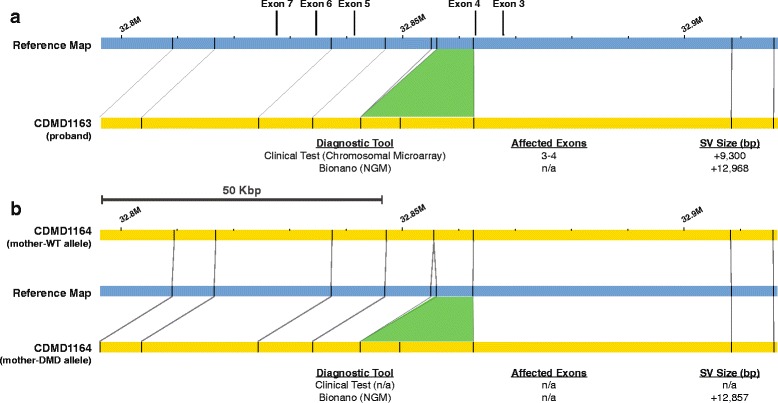
NGM identified a 13-Kbp insertion in a DMD patient and his biological mother. **a** *Top*: visual representation of the insertion (*green*) between the reference (*blue*) and patient (*yellow*) maps. *Bottom*: insertion size identified in the proband by chromosomal microarray and by NGM platform. **b** *Top*: the normal wild type allele of the mother (*yellow*) can be seen above reference (*blue*) where all nicking sites align to reference map. This is in contrary to the second allele of the mother (*yellow*) containing the insertion shown below the reference (*blue*) map. Maps were generated using Nt.BspQI nicking endonuclease

Proband CDMD1163 was diagnosed clinically with a duplication of exons 3–4 by chromosomal microarray and the status of the duplication in the mother (CDMD1164) was unknown. NGM identified a 12.9-Kbp insertion in the proband (CDMD1163) that included exons 3–4 of the *DMD* gene, that was also present in a heterozygous state in the mother (CDMD1164) (Fig. 7 a, b). Since the carrier status of the mother had not been determined clinically, we validated the NGM findings using qPCR (Additional file 2: Figure S1).

**Fig. 8:**
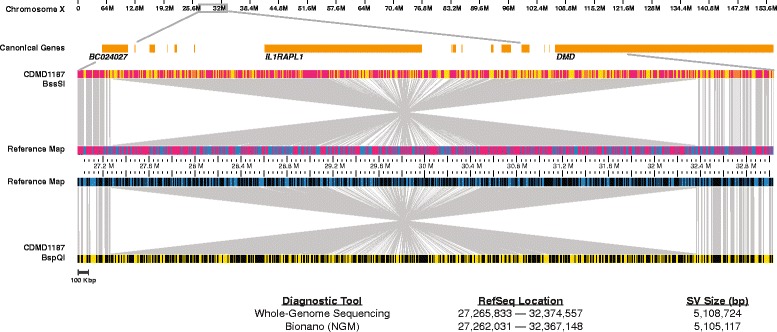
NGM identified a 5.1-Mbp inversion disrupting *DMD. Top*: X chromosome and Ref-Seq genes (*orange*) present in the magnified region. Visual representation of the inversion where the middle section of the reference (*blue*) and patient (*yellow*) maps have inverted alignments. The sample maps were generated using Nb.BssSI (*top*) and Nt.BspQI (*bottom*) endonucleases. Nicked sites are represented by *red* (Nb.BssSI) or *black* (Nt.BspQI) *vertical lines* in the middle reference and top/bottom sample maps

One of the defining features of Bionano’s NGM system is its capability to identify inversions, which cannot be detected with chromosomal microarrays. One of the patients in our DMD cohort (CDMD1187) had been clinically diagnosed with DMD by muscle biopsy, but neither MLPA, PCR sequencing of all 79 exons, nor exome sequencing revealed pathogenic mutations. In a parallel effort to the NGM work reported here, WGS revealed a large 5.1-Mbp inversion in intron 38 of *DMD* that disrupted the RNA splicing starting from exon 38, confirmed by transcriptome sequencing (data not shown). Because WGS was performed as part of a research study, appropriate PCR primers for this unique mutation were developed within the UCLA Orphan Disease Testing Center to allow a simple PCR/sequencing diagnostic useful for detecting carrier status for patient’s first degree female relatives. This sample provided an opportunity to assess the ability of NGM to robustly identify inversions. We performed NGM using the Saphyr instrument with two nicking endonucleases (Nt.BspQI and Nb.BssSI) generating 114x and 66x effective coverage, respectively. NGM identified the 5.1-Mbp inversion with breakpoints mapped at high confidence within 3–7 Kbp of the exact breakpoint determined by WGS (Fig. 8).

Here, we demonstrate that NGM can identify SVs in the *DMD* gene in both hemizygous and heterozygous states in size ranges of 13 Kbp to 5.1 Mbp. However, in considering how the method may be applied to the diagnosis of rare genetic diseases, it is important to recognize that each genome studied here had many other SV outside of the DMD locus (data not shown). Some of the other SVs are likely due to errors in the common reference of the genome, false positives, or are too common to be classified as causal for rare Mendelian diseases. We suggest that future studies utilizing NGM for identification of novel structural variants involved in disease filter variants using the Database of Genomic Variants (DGV) or other type of repository of SV for maximal removal of common, likely benign structural variants.

## Discussion

For many years in genetic diagnostics, the primary focus has been on SNVs using Sanger sequencing. With the advent and clinical implementation of exome sequencing, the rate of diagnosis has significantly increased. However, only about 30% of all cases referred for clinical exome sequencing result in a clear molecular diagnosis [1, 2]. It is likely that a substantial portion of these missed diagnoses is due to the fact that the vast majority of the human genome is not observed when performing exome sequencing. While point mutations in intergenic and intronic regions can result in disruption of expression or reading frame of a given mRNA, SV are an important category of variation insufficiently observed from current clinical testing. WGS of short fragments in the range of 300–400 bp can sensitively reveal intergenic and intronic SNVs and small INDELs, but in regions of the genome that are duplicated or with higher order repeats, the mapping of reads prevents discovery of SV. These limitations can be overcome by NGM, which identifies large structural variants in human genomes with high confidence.

The sizes of the insertions and deletions that can be identified using NGM are dependent upon the length of the labeled DNA molecules and the frequency of nicking endonuclease recognition sites on the + and – strands of the genome. If nick sites on complementary strands are too close, both strands of DNA are nicked, which could result in a dsDNA break and compromised mapping at that location. If long DNA molecules are broken during purification or nicking, the effective haplotyping is also compromised. Both of these issues may be improved by slower DNA mixing techniques that preserve DNA integrity and reduce the likelihood of dsDNA breakage during nick extension and dsDNA labeling. The DNA purification protocol currently implemented requires sample preparation in agarose, allowing for less physical shearing to preserve the length of DNA molecules. As shown in the mapping of CDMD1187, a second endonuclease can improve breakpoint resolution and provide a more uniform genome coverage due to the presence of additional restriction sites. Here, we used two endonucleases (Nt.BspQI and Nb.BssSI) for the CDMD1187 sample, which allowed better genome coverage and more specific breakpoint resolution. Improvements in the sizing of the linearized DNA fragments in nanochannels may also improve resolution.

NGM has the capacity to replace both MLPA and chromosomal microarrays in the clinical setting. It provides a number of key advantages. Compared to MLPA, it is genome-wide and provides both the order and orientation of structural variants. Compared to chromosomal microarray, in addition to duplications, deletions, and translocations that result in DNA material loss or addition, NGM detects balanced events, such as inversions and balanced translocations as well as much smaller kb-size SV. With regard to NGS with base-pair resolution, NGM provides higher sensitivity for large structural variants with better false-positive and false-negative rates [4, 5, 9]. The current turnaround time for a single sample with dual endonuclease genome assembly is approximately 1–2 weeks, which is well within the time frame of most other clinical genetic tests. Associated costs are comparable to the current costs of WGS.

Long-read technologies, such as the one demonstrated here, offer a more complete representation of a given human diploid genome that complements and augments data from short-read technology. NGM technology has the promise to observe transposon-mediated pathogenic mutations, even though transposons are highly repetitive in the human genome. Further, much of the known SVs can be mediated through local sequence homology between repetitive portions of the genome. Thus, observing these SV by long reads should provide greater resolution of SV throughout the genome. The interpretation of pathogenicity of non-coding variants will present challenges; however, with larger databases of SV and the concomitant effect on gene expression, the scientific community will be able to solve a larger fraction of undiagnosed genetic diseases. While we could sensitively detect the *DMD* mutations here, more broad usage may require better tools to determine variant pathogenicity in unknown genes. Next steps are to use this technology in cases where the location of the pathogenic variants is not known and attempt to identify them in a variety of disease types.

## Conclusions

We used a cohort of patients diagnosed with DMD with known structural variants in the *DMD* gene to validate the capability of the NGM platform to accurately identify large deletions, insertions, and inversions in the hemizygous and heterozygous states. We have had a 100% concordance rate with clinical tests in this small cohort using NGM, indicating the clinical utility of the method. NGM promises to help further our understanding of gene regulatory elements in the genome and of how SNV and SV in these regions may affect gene regulation.

## Additional files

Additional file 1: Table S1. Primers used for qPCR validations of structural variants. List of primer sequences and corresponding annealing temperatures. Upper case letters indicate that the primer sequence is within the exon. (PDF 40 kb)

Additional file 2: Figure S1. *DMD* exons 3–4 duplication validation in the CDMD1163 case. Exons 2 and 5 are present in single copies on the X chromosome in the proband (*light green*). The mother (CDMD1164), who has 2 X chromosomes, has 2x more DNA (*dark green*) indicated by fold change (FC). Exons 3 and 4 are duplicated in the proband and the mother is a carrier - DNA amount in proband (2 copies) is approximately 1.5x lower than in mother (3 copies) consistent with a duplication being present in both. (PDF 153 kb)

## Abbreviations

DMD: Duchenne muscular dystrophy
dsDNA: Double-stranded DNA
INDEL: Small insertions and deletions
MLPA: Multiplex ligation-dependent probe amplification
NGM: Next-generation mapping
PCR: Polymerase chain reaction
SNV: Single nucleotide variant
SV: Structural variants
WGS: Whole-genome sequencing
