# Supplementary material for: Next-generation mapping: a novel approach for detection of pathogenic structural variants with a potential utility in clinical diagnosis
Source: Genome Med. 2017 Oct 25;9:90. doi: 10.1186/s13073-017-0479-0 (PMC5655859; doi:10.1186/s13073-017-0479-0)
Supplement: Supplementary file 1 — Primers used for qPCR validations of structural variants. List of primer sequences and corresponding annealing temperatures. Upper case letters indicate that the primer sequence is within the exon. (PDF 40 kb) [file 13073_2017_479_MOESM1_ESM.pdf]

Exon	Forward Primer	Reverse Primer	Annealing Temperature
2	tgcattttagATGAAAGAGAAGATG	aaaacggatttttaagatacacagg	60
3	tggaagtgtgctttgttaaattg	tgccaaatgaaaatcatacgag	60
4	gtaccaggccaaggacaattag	aagccctcactcaaacatgaag	60
5	cccctttctttaacagGTTGATT	catttgtttcacacgtcaagg	60
