# Supplementary figures and images for: Next-generation mapping: a novel approach for detection of pathogenic structural variants with a potential utility in clinical diagnosis
Source: Genome Med. 2017 Oct 25;9:90. doi: 10.1186/s13073-017-0479-0 (PMC5655859; doi:10.1186/s13073-017-0479-0)

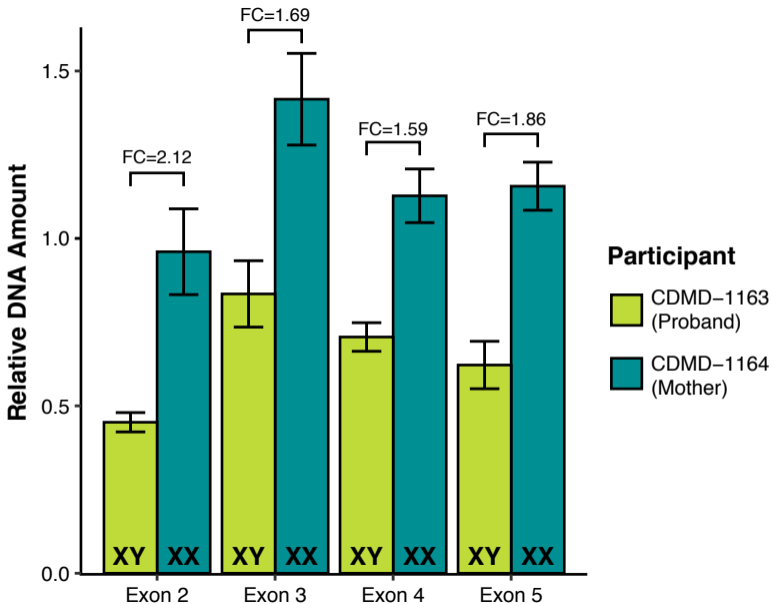

Supplement: Supplementary file 2 — DMD exons 3–4 duplication validation in the CDMD1163 case. Exons 2 and 5 are present in single copies on the X chromosome in the proband (light green). The mother (CDMD1164), who has 2 X chromosomes, has 2x more DNA (dark green) indicated by fold change (FC). Exons 3 and 4 are duplicated in the proband and the mother is a carrier - DNA amount in proband (2 copies) is approximately 1.5x lower than in mother (3 copies) consistent with a duplication being present in both. (PDF 153 kb) [file 13073_2017_479_MOESM2_ESM.pdf]
